# Influence of Sociospatial determinants on knowledge, attitudes and practices related to the plague in a population living in endemic areas in the central highlands, Madagascar

**DOI:** 10.1186/s12889-021-11101-3

**Published:** 2021-06-09

**Authors:** Sitraka Rakotosamimanana, Feno Jacob Rakotoarimanana, Vaomalala Raharimanga, François Taglioni, Josélyne Ramamonjisoa, Rindra Vatosoa Randremanana, Minoarisoa Rajerison, Fanjasoa Rakotomanana

**Affiliations:** 1 Institut Pasteur de Madagascar, 101 Antananarivo, Madagascar; 2 Université d’Antananarivo, 101 Antananarivo, Madagascar; 3 Université de La Réunion, Saint-Denis, France; 4 ACCESS Health Program, Management Sciences for Health, Antananarivo, Madagascar; 5 UMR Prodig, Paris, France

**Keywords:** Plague, Madagascar, Central highlands, KAP scores, Sociospatial determinants

## Abstract

**Background:**

Plague is endemic to the central highlands of Madagascar. Sporadic human cases or outbreaks can occur annually in these areas. In Madagascar, the associations between endemicity and the knowledge, attitudes and practices (KAP) of the population with regard to this disease remain poorly documented. The aim of this study was to assess KAP related to plague among the population living in the central highlands.

**Methods:**

A cross-sectional survey was conducted in the general population from June to August 2017. Based on the reported cases of plague between 2006 and 2015 in two central highland districts, a KAP questionnaire was administered in the population. Based on the proportion of correct answers provided by respondents, KAP scores were classified into three KAP categories: low (< Mean - SD), medium (Mean ± SD) and good (> Mean + SD). Multivariate analyses were performed to determine the associations between population KAP scores related to plague and sociodemographic and epidemiological factors. In addition, individual interviews and focus groups with health professionals were conducted to assess plague perception.

**Results:**

A total of 597 individuals participated in the survey; 20% (*n* = 119) had a good KAP score, 62% (*n* = 370) a medium KAP score and 18% (*n* = 108) a low KAP score. Among the 119 respondents with good KAP scores, 80% (*n* = 95) resided in Ambositra district, and 20% (*n* = 24) resided in Tsiroanomandidy district. According to the health professionals in the two districts, populations in endemic areas are well aware of the plague. There were significant associations (*p* <  0.05) of not owning a mobile phone, having no contact with a former plague case, and living in Tsiroanomandidy district with a lower KAP score.

**Conclusion:**

The results of the study showed the need to adapt plague control interventions to the local context to allow a better allocation of human and financial resources. Doing so would minimize delays in patient management care and increase community resilience to plague epidemics.

**Supplementary Information:**

The online version contains supplementary material available at 10.1186/s12889-021-11101-3.

## Background

Plague is a neglected infectious disease. This zoonotic disease can affect humans. There are plague reservoirs worldwide, but only a few regions report human plague cases. In 2015, 75% of human cases were reported in Africa (3248 worldwide recorded cases) [1].

In Madagascar, plague was introduced in 1898. Since 1921, it has been endemic in areas more than 800 m above sea level in the central and northern highlands [2–4]. Human plague occurrences are not limited to the Malagasy highlands. Human cases occur sporadically in other regions of Madagascar [5, 6]. Due to the annual occurrence of human cases and deaths, plague remains a public health problem in Madagascar [7, 8]. Every year, approximately 400 cases of human plague are reported in Madagascar [7, 8]. Exceptionally, in 2017, an urban plague outbreak with a majority of pneumonic plague cases had many victims; 209 deaths out of 2417 cases were reported during this season, and it affected major urban cities [8, 9]. Although endemic to Madagascar since the end of the nineteenth century, knowledge, attitudes and perceptions about plague remain understudied. These are contributing factors that could potentially be linked to the annual increase in plague cases in endemic areas. It could also be a source of delays in care seeking.

The impact of ecological and environmental factors (climate, vegetation and altitude) on the maintenance of plague or their link to the occurrence of human plague cases is known [1, 7]. However, the role of human behaviour or population perceptions associated with plague or other infectious diseases has rarely been explored [10].

One of the main obstacles to plague control is the misunderstanding of plague characteristics by the population. Understanding the relationships between human plague occurrence and disease awareness could be crucial in making decisions for plague control [10, 11]. Data collected by a knowledge, attitudes and practices (KAP) survey were used to assess the knowledge, beliefs and perceptions of individuals and to understand their attitudes and practices regarding public health problems [12]. A KAP study allows for the identification of gaps in the various education or awareness programs in relation to a multitude of public health themes [12–24]. While similar studies have been conducted in endemic areas, such as Uganda [17, 22] and Zambia [18, 25], associations between endemic areas and people’s knowledge of the disease remain underdocumented.

The aims of the study are: (i) to assess the population’s KAPs on plague; and (ii) to determine the influence of epidemiological features and sociospatial determinants on the population’s KAP scores in two central highland districts.

## Methods

### Study district

Two districts were selected: Ambositra and Tsiroanomandidy. These two districts were chosen because they belong to the central highlands region and because the Ambositra district was a very active endemic plague area in the 2000s [26], and the Tsiroanomandidy district has been one of the most active plague districts until now [4].

**Fig. 1:**
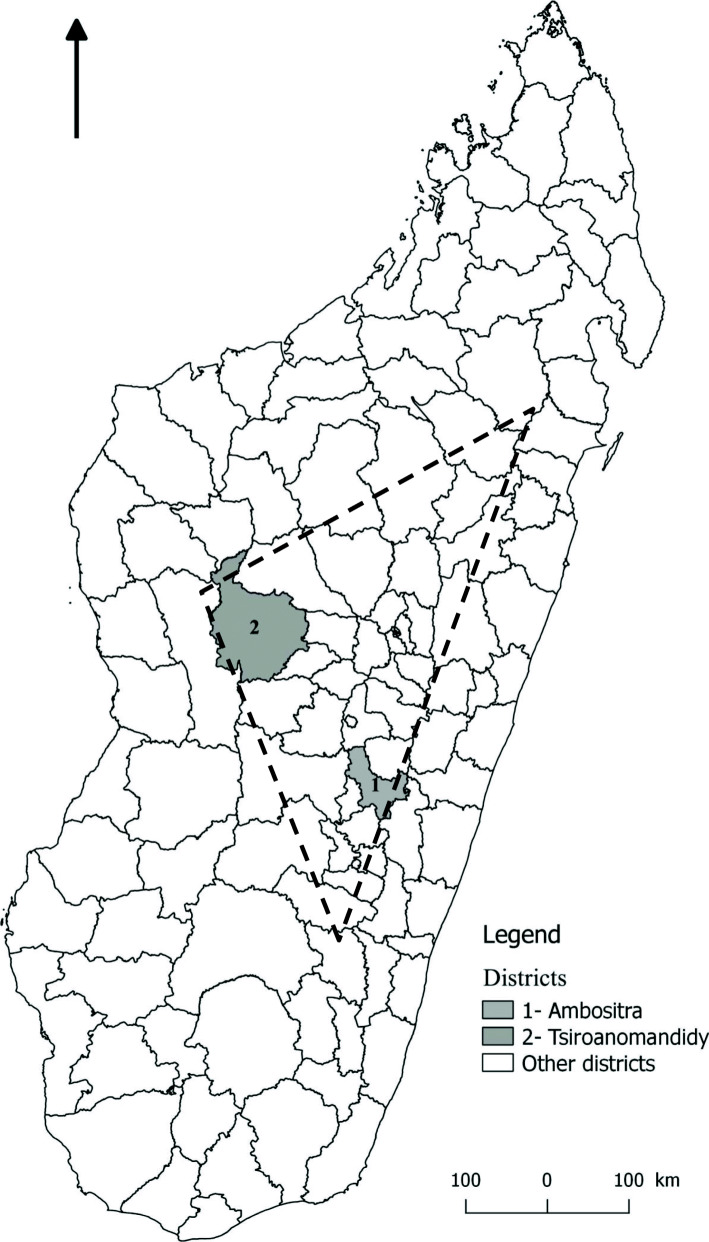
Locations of the studied districts - The map was performed by S. Rakotosamimanana using free and open source Quantum GIS (QGIS) 3.4® software

The selection of studied sites was based on the number of plague cases reported between 2006 and 2015 in the database of the Central Laboratory for Plague hosted at the *Institut Pasteur de Madagascar* (IPM), Antananarivo, Madagascar. From this database, two sites in the central highlands were selected for the KAP survey (Fig. 1). (i) Tsiroanomandidy district, in the Middle West of Madagascar. It is located 215 km northwest of Antananarivo, the capital. It counts 17 municipalities. It is an active focus with altitudes ranging from 800 to 1500 m. Human plague cases are reported annually in this district. This district recorded an average of 40 cases per year with peaks during the selected period. (ii) The Ambositra district on the southern axis of Madagascar. Located 255 km south of Antananarivo, Ambositra has 23 municipalities. It is an active plague focus where altitudes range from 700 to 1000 m. In the 2000s, it was one of the districts with the highest incidences of human plague in Madagascar. Between the selected periods, some years without human plague cases were recorded. On average, 10 cases per year were reported between 2006 and 2015 in this district.

### Study design

This study was a descriptive, exploratory mixed cross-sectional study. It was conducted in the general population from the municipalities of two endemic plague districts in the central highlands: Ambositra and Tsiroanomandidy. To assess KAPs towards plague of the general population, a quantitative survey was conducted. In addition to the KAP quantitative survey, we performed a qualitative study of health professionals’ perceptions of plague.

### Distribution of plague cases in study districts

A database was compiled with all suspected, probable and confirmed cases of human plague reported in Madagascar’s health facilities. As a first step, a mapping of the distribution of plague cases by year at different administrative scales (district, municipality) was performed. To classify municipalities for investigation, we used information from plague database, including individual address, clinical forms of plague (bubonic, pulmonary or septicaemic plague), category of case and the health facility of the reported case.

The municipalities were classified according to the presence or absence of cases of plague. Probable and confirmed cases were considered in this study. Based on WHO recommendations in 2006, a probable case was defined as a clinically suspected case with a positive rapid diagnostic test or positive molecular biology and culture negative or not performed, and a confirmed case was defined as a suspected case with a positive rapid diagnosis testing or positive molecular biology and positive culture [9, 27]. The municipalities to be surveyed were randomly selected according to the category of municipality. Depending on the presence or absence of cases per municipality, municipalities were classified into two categories: (i) municipalities with a presence of cases (reported at least one case of plague during the selected period); and (ii) municipalities without cases (no cases recorded during the selected period). Among the 17 municipalities in Tsiroanomandidy district, only one municipality did not report any cases of plague between 2006 and 2015. A total of 18 of 23 municipalities reported cases of plague in the Ambositra district during the study period.

### KAP study

**Fig. 2:**
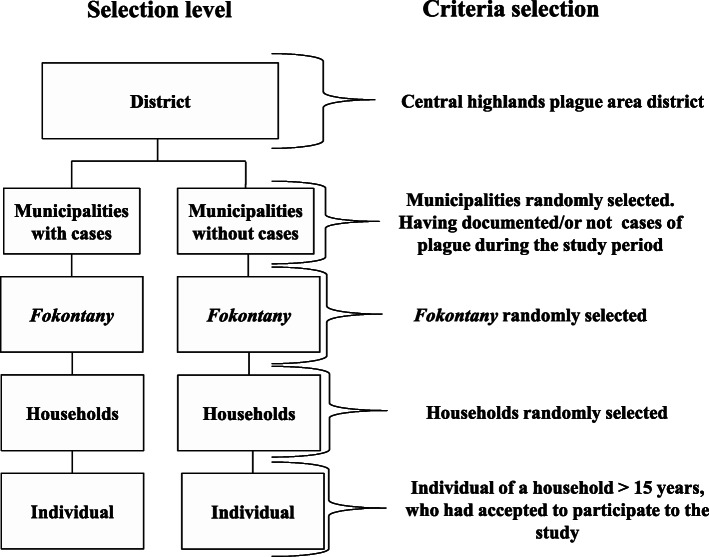
Flowchart of study structure

The questionnaires were pretested in another endemic district. Adjustments and improvements were performed after this test phase. The data from the pretest survey were not included in the final analysis. The surveys were conducted between June and August 2017. A two-step selection was performed at the municipality and “*fokontany*” levels (the smallest administrative unit in Madagascar) (Fig. 2). The municipalities to be investigated were randomly selected for each district and according to whether the municipalities reported plague cases during the study period. Then, we randomly selected *fokontany* per municipality category for field investigation. For Ambositra, 7 municipalities were investigated, including 5 municipalities with cases and 2 municipalities without cases during the study period, among which 11 *fokontany* were visited. For the case of Tsiroanomandidy, a total of 10 *fokontany* were visited. These *fokontany* are part of 7 municipalities, including 6 municipalities with cases and one municipality without cases. Approximately 30 people per site were randomly selected and surveyed. With two consenting persons per household, we estimated 20 households to be visited per *fokontany.* Households were randomly selected to obtain up to thirty investigated individuals. Household members were listed, and the questionnaire was administered to a maximum of two participants per household. The participants were randomly selected if the households presented more than two members. The criteria for selecting the subjects were as follows: individuals aged 15 years old or older at the time of the survey who agreed to participate in the study and who signed the informed consent form. If household members were absent or unwilling to participate, another household was randomly selected.

### Data collection

The questionnaire was based on the WHO KAP methodology on tuberculosis [28] and adapted to the Malagasy context (Additional file 1). The questionnaire was administered in the Malagasy native language. It was designed to measure the following constructs: (i) general information; (ii) population knowledge on plague; (iii) attitudes adopted in the case of illness; and (iv) general practices in the case of care seeking.

The general information section contained all of the personal information and sociodemographic characteristics of the interviewed individuals. The section on the knowledge about plague contained 14 questions, including the number of known types of plague, known forms of plague, symptoms of plague, contagious nature of plague, mode of transmission of plague, lethal nature of plague, time at which plague can be fatal after the first signs appear, existence of a treatment for plague, treatments for plague, place of access to care and treatment. The attitudes in the case of illness section contained 5 questions. General practices in case of care seeking presented 6 questions.

### Data analysis

#### KAP scoring

KAP scores were assigned to respondents by individual scores based on the literature and adapted to plague items. Scores were assigned based on the proportion of correct answers provided by respondents to the total possible correct answers [13, 18, 23–25, 29]. The KAP questionnaire (Additional file 1.) had 3 components for a total of 39 (100%) possible points: 24 points for the knowledge component (61% of possible correct answers), 12 points for the attitude component (30% of possible correct answers), and 3 points for the practices component (7% of possible correct answers). The combined KAP scores (combined knowledge, attitudes and practices) were classified into three categories according to the scores obtained by respondents: low KAP (< Mean - 1 SD), medium KAP (Mean ± 1 SD) and good KAP (> Mean ± 1 SD) [16, 19].

### Statistical analysis

The data analysis was performed using Microsoft Excel 2010® software and Stata software, version 13®. Descriptive analysis was used to summarize the characteristics of the respondents and their KAP scores on plague. We fitted univariate ordinal logistic regressions for each explanatory variable. Explanatory variables with *p* ≤ 0.2 in the univariate analysis were considered further in the multivariate analysis. Variables that could influence the KAP score were also included in the final model based on our assumptions.

Independent variables included in the model were: (i) sociospatial characteristics: age (continuous variable), sex (binary variable), level of education (categorical variable), telephone ownership (binary variable), and district of residence (binary variable); and (ii) characteristics related to history of plague and/or epidemiological status of the investigated localities (binary variable): contact with a former plague case (categorical variable) and municipality category (presence/absence of plague cases, binary variable). KAP scores and ages were transformed into categorical variables for analysis.

A backwards elimination process was used to build the final multivariate models for each of the outcome variables. In the final model, the statistical significance level was set at *p* <  0.05.

### Qualitative study of the perception of plague among health actors

A qualitative study of health professionals’ perceptions of plague was performed. The study was conducted on the basis of individual interviews and focus groups with health professionals of all ages, genders and functions. In each of the two municipalities, two facilities per district were selected for the qualitative study. Three semidirective individual interviews and one focus group, with a total of 10 participants, were conducted in the facilities of the Basic Health Centre (CSB) of the two central highlands districts. Two CSBs in Ambositra district were surveyed with 8 health actors participating in the focus group and interview: two doctors, a nurse, three midwives, a paramedic trainee and a health care assistant. Two individual interviews were conducted in two CSBs in the Tsiroanomandidy district with two participating doctors. The average duration of an interview (individual or focus group) was 34 min. Facilities were selected from municipalities investigated during the KAP study depending on the availability of medical staff for interviews. One focus group and three individual interviews were conducted. A pre-established interview template was used for both types of interviews. The framework had three main components: a knowledge component on health professionals on plague (symptoms, clinical forms, transmission modes, treatment, etc.); a component on their perception of communities’ attitudes in the case of plague occurrence; and a component on their perception of population practices in the case of plague occurrence. After the participants consented, focus groups and individual interviews were recorded using a dictaphone and then transcribed and translated.

### Data analysis

A manually thematic method was used to analyse the data collected from individual interviews and focus groups. Information was categorized, coded by component categories and manually analysed.

### Ethical considerations

The study received approval from the Ethics Committee board of the Malagasy Ministry of Public Health (n#50 MINSANP/CE of 27 April 2016). All of the surveys were systematically preceded by an information session on the processes and purposes of the study. All of the participants signed an informed consent form. For individuals younger than 16 years old who agreed to participate in the study, the guardian or a parent provided consent and signed the informed consent form. Each participant was allowed to decline or to leave the study at any time.

## Results

A total of 597 individuals were interviewed, of whom approximately 61% (*n* = 364) were women and 39% (*n* = 233) were men, with a sex ratio of 0.64 (male/female). The median age was 36 years old (ranging from 15 to 80). Nearly 25% (*n* = 148) of the respondents were aged between 15 and 24 years old. Approximately 7% (*n* = 40) did not have any formal education, approximately 52% (*n* = 316) of respondents had a primary school education, nearly 38% (*n* = 225) had a level equivalent to the secondary school level, and 3% (*n* = 16) had higher educations. A total of 340 individuals interviewed (57%) did not have a mobile phone. The proportion of individuals who had contact with a person who had already contracted plague was approximately 11% (*n* = 64); nearly 45% (*n* = 29) of them were family members. The sociodemographic features of the study population are shown in Table 1.

**Table 1:** Sociodemographic features of the study participants

Variables	Number	Percentage (%)
**Age (Mean ± SD**), **Years**	**38 ± 15**	
**Age group (years)**		
15–24	148	24.8
25–34	127	21.3
35–44	126	21.1
45–54	89	14.9
> 55	107	17.9
**Sex**		
Female	364	61
Male	233	39
**Sex ratio (M**: **F)**	**0.64 (233: 364)**	
**Educational level**		
None	40	6.7
Primary	316	52.9
Secondary	225	37.7
High	16	2.7
**Mobile phone owning**		
Yes	257	43.1
No	340	56.9
**Contact with former plague case**		
Yes	64	10.7
No	530	88.8
Do not know	3	0.5
**Residence district**		
Ambositra	325	54.4
Tsiroanomandidy	272	45.6
**Category of the investigated municipality**		
Without case	82	13.7
With case	515	86.3

### Knowledge about plague

For the question “Have you ever heard about plague?”, 97% (*n* = 580) of the respondents indicated already having heard about it. The 3% (*n* = 17) who had never heard about it resided in Ambositra district. Approximately 38% (*n* = 227) of individuals knew only of the bubonic form. Only 1 % knew only the pneumonic or septicaemic form. Among individuals who mentioned at least two plague forms, 30% (*n* = 174) knew the bubonic and pneumonic forms, 4% (*n* = 23) knew the bubonic and septicaemic forms, and 5% (*n* = 32) of individuals cited all three forms (bubonic, pneumonic and septicaemic). Approximately 20% (*n* = 115) could not list any of the plague forms.

Regarding plague symptoms, 84% (*n* = 490) of respondents were able to cite at least one sign that might suggest plague, such as fever or the presence of buboes on the body.

The contagious nature of plague was well known, and 95% (*n* = 551) of respondents answered “yes” to the question “Is plague contagious?”. One percent (*n* = 4) answered “no” to the question, and 4% (*n* = 25) did not know whether plague was contagious.

Regarding the mode of transmission, 54% (*n* = 315) of respondents said that plague was transmitted by the bite of a rat flea, and 21% (*n* = 122) cited other modes of transmission, such as mosquito bites, lack of environmental hygiene, and objects contaminated by patients. Ninety-six percent (*n* = 556) of respondents mentioned that plague was fatal, and 67% (*n* = 387) stated that people can die from plague in less than 3 days. Ninety-two percent (*n* = 532) said that a drug or treatment for plague existed, 3% (*n* = 16) said that treatment did not exist, and 5% (*n* = 32) did not know whether treatment existed.

### In the event of illness, attitudes towards others

In the event of illness, nearly 97% (*n* = 576) of respondents said they shared what happens to those around them. Approximately 90% (*n* = 537) said they undertook specific measures to protect those around them in case of illness. Some, nearly 35% (189/537), said they went to the nearest health facility, approximately 23% (124/537) went to the doctor, and approximately 13% (68/537) said they did both. A total of 26% (*n* = 129) mentioned that they undertook other types of measures, such as providing medicines to those around them, isolating themselves, using medicinal plants or not undertaking specific measures.

### Practices for seeking care in cases of plague or other diseases

When the participants were asked, “In case of signs that might suggest plague (fever, buboes...), would you go to see a doctor?”, 67% (*n* = 403) said they would go to a doctor if signs that might suggest plague appeared. Approximately 28% (*n* = 165) reported indecision if signs of plague appeared. Approximately 5% (*n* = 29) said they did not go to a doctor if they presented with plague symptoms.

In the case of other diseases, the majority of respondents (81%) (*n* = 483) said they went to the hospital or to a health facility; approximately 14% (*n* = 84) reported both hospitals and health facilities. Only 5% (*n* = 30) reported going neither to a health facility nor to a hospital.

### Evaluation of KAP scores

**Fig. 3:**
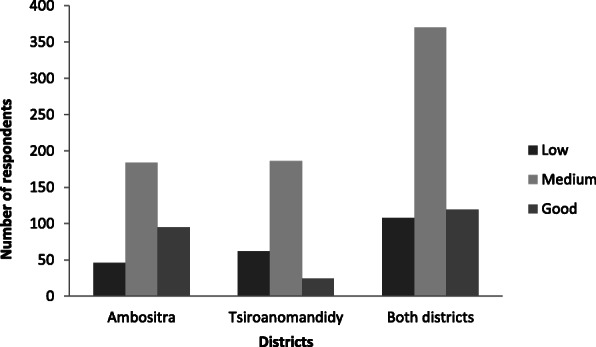
Distribution of KAP scores by studied district

For all of the survey participants (*N* = 597), after assigning KAP scores, the average KAP score was 19.4 with a standard deviation (SD) of 6.3. Approximately 18% (108/597) had a low KAP score, 62% (370/597) a medium KAP score and 20% (119/597) a good KAP score (Fig. 3).

For the individuals surveyed in Ambositra (*n* = 325), the average KAP score was 20.94 with an SD of 6.68. Approximately 14% (46/325) had a low KAP score, 57% (184/325) had a medium KAP score, and 29% (95/325) had a high KAP score.

For the individuals interviewed at Tsiroanomandidy (*n* = 272), the average KAP score was 17.52, and the SD was 5.22. Approximately 23% (62/272) had a low KAP score, nearly 68% (186/272) had a medium KAP score, and nearly 9% (24/272) had a high KAP score.

### Segregating KAP score

Following are the segregated KAP scores for the two districts.

### Knowledge score

For both districts, the average knowledge score was 11.84 with an SD of 4.35. For the Ambositra district, the average knowledge score was 12.35 (SD, 4.79). For Tsiroanomandidy, the average knowledge score was 11.25 (SD, 3.68).

### Attitudes score

The average attitudes score for both districts was 5.77 with an SD of 3.3. By district, the average attitude score was 6.62 (SD, 3.09) for Ambositra and 4.75 (SD, 3.26) for Tsiroanomandidy.

### Practices score

The average practices score was 1.76 (SD, 0.68) for both districts. For Ambositra, the average practices score was 1.97 (SD, 0.66). For Tsiroanomandidy it was 1.52 (SD, 0.61).

### Multivariate analysis

We found a statistically significant association of the four variables with the KAP scores of individuals (Table 2): (i) mobile phone ownership; (ii) contact with a former plague case; (iii) district of residence; and (iv) status of the municipality (absence/presence of plague case). Our results showed that the possibility of having a higher KAP score (average or good) decreased for an individual without a telephone [adjusted odds ratio ORa = 0.64 (95% CI 0.46–0.90), *p* = 0.009] who had never been in contact with a suspected case of plague [ORa = 0.42 (95% CI 0.25, 0.71), *p* = 0.001] and who lived in Tsiroanomandidy [ORa = 0.37 (95% IC 0.26–0.52), *p* <  0.001].

**Table 2:** Associations between KAP scores and other variables (multivariate analysis)

Variables	OR ***adjusted***	95% CI	***p-value***
**Mobile phone owning**			
Yes	–	–	*–*
No	0.64	0.46–0.9	*< 0.05*
**Contact with former plague case**			
Yes	–	–	*–*
No	0.42	0.25–0.71	*< 0.05*
Don’t know	0.67	0.05–7.12	*0.7*
**Residence district**			
Ambositra	–	–	*–*
Tsiroanomandidy	0.37	0.26–0.52	*< 0.05*
**Category of investigated municipality**			
Without case	–	–	*–*
With case	2.13	1.32–3.45	*< 0.05*

Conversely, residing in municipalities where plague cases were reported between 2006 and 2015 increased an individual’s possibility of having a higher KAP score [ORa = 2.13 (95% CI 1.32–3.45), *p* = 0.002].

### Health professionals’ perceptions of plague

A total of four focus groups and individual interviews were conducted: two in the Tsiroanomandidy district and two in the Ambositra district. Most of the interviewed participants were able to identify two clinical forms of plague: bubonic plague and pulmonary plague. Septicaemic plague was not frequently cited, and its symptoms are not well understood by health professionals. Dirtiness, poor hygiene, fleas and rats were identified by participants as the sources of plague. Airborne transmission of pneumonic plague was also mentioned by all of the respondents. The contagious nature and that plague is a fatal disease without prompt treatment were also mentioned by all of the participants.

### Health professionals’ perceptions of population knowledge about plague

Here are some excerpts from interviews on health professionals’ perceptions of population knowledge about plague:

“People here already know [plague] because many sensitizations have already been conducted”.

“(...) community health workers are already quite experienced about plague (...) their knowledge of symptoms is already quite good”.

### Health professionals’ perceptions of community attitudes towards plague

“Being in a plague area, we have adopted a strategy to provide care rapidly”.

“As soon as they suspect plague, they go to the hospital right away”.

“(...) people are afraid of it! They are afraid of plague because people have always known that it is a terrible disease (...)”.

“In fact, we are in a plague focus. People are almost aware of plague; in this case, it is plague, and they are no longer going to traditional healers. As soon as people notice something suspicious, they say right away: it is plague, doctor”.

## Discussion

The inhabitants of the two districts had significantly different KAP scores. Most people in these two districts have already heard about plague. In the case of plague outbreaks, interventions were led by local or national health authorities, as in Uganda [17, 20]. Although these two districts are located in plague-endemic areas, the epidemiological contexts are different from one another. Tsiroanomandidy district documented an annual occurrence of human plague cases between 2006 and 2015, and Ambositra district experienced a quiet period in 2010.

According to our results, bubonic and pneumonic forms were known by respondents. Septicaemic plague is not well known to health workers and populations, likely because of the nonspecificity of its clinical symptoms and its rarity compared to other forms of plague in Madagascar [30, 31]. In both districts, the population often associated the mode of plague transmission with rats or dirtiness, although transmission of the disease by flea bites and/or by air was also mentioned, unlike in parts of Africa such as Zambia, where rats and fleas were more commonly mentioned by respondents [18]. Indeed, in Petauke, Zambia, two plague outbreaks occurred in 2001, and a study showed that bubonic plague – in particular the identification of the role of rats and fleas in plague transmission – was known by the populations.

In Madagascar, good or medium KAP scores could be explained by the almost permanent presence of plague control interventions in endemic areas of the central highlands. The population living in Ambositra had higher KAP scores than the population living in Tsiroanomandidy district. For all of the individuals interviewed, bubonic plague was the best-known form of plague. It is the most common clinical form of human plague in Madagascar [3–5].

The probability of having a high KAP score was associated with the possession of a telecommunications tool, likely because information about plague and health facts in general can be quickly circulated by rumour, word of mouth or telephone messages (Table 2).

Being in contact with a former plague case increased the chances that an individual would have a higher KAP score compared to another individual who had not been in contact. The assumption of having been in contact with a person suspected of plague would have prompted close families to document and inform themselves to better understand this disease. This outcome could be explained by the assumption that knowledge about plague among individuals is based on the experience of plague in the family or the environment. Conversely, community awareness campaigns and responses targeting living populations, neighbours or family members of former cases in a locality where plague cases are reported could come into play. In Madagascar, for each case of plague reported in a locality, the case and its surroundings systematically benefit from an awareness session, insecticide application and contact chemoprophylaxis, according to the recommendations of the national plague program.

We can observe that the level of education was not associated with a high KAP score for this sample. This outcome might be explained by information about the disease being incomplete during the school years, although plague has been included in the school curriculum since primary school. In addition, there is the likelihood of recall bias. Populations might pay more attention to the health messages conveyed in the mass media or during awareness or information campaigns on plague.

Living in Tsiroanomandidy could be associated with the likelihood of having a low KAP score for plague. Tsiroanomandidy is a large district in area (10,199 km^2^) compared to district Ambositra district (3161 km^2^), which is less densely populated (33 inhabitants per km^2^ versus 95 for Ambositra according to a Malagasy National Statistics Institute projection in 2015) but also a more rural district. Indeed, awareness and information measures could not have covered the entire Tsiroanomandidy district since some localities are isolated and difficult to access. Compared to the population of other municipalities in the two districts of Madagascar that did not document plague cases between 2006 and 2015, populations living in municipalities where plague cases were reported during the same period had a good or medium KAP score. These findings suggest that the populations of municipalities that reported localized plague cases in the central highlands have higher KAP scores but also greater knowledge of plague. These findings cannot be extrapolated to all populations of endemic areas in Madagascar. The KAP score on plague could be influenced by the epidemiological context of localities, such as Uganda, where populations living in plague outbreak areas had a better knowledge of the disease [17].

In this study, we treated the responses of individuals from the same household independently for the KAP investigations. This fact is a limitation of the study since it could constitute a bias. Indeed, members of the same household are likely to have the same perceptions about the disease. However, a major constraint during the KAP survey was that, in some *fokontany* of both districts, there were few households available for the survey. It was therefore sometimes necessary to administer the questionnaire to a maximum of two people in the same household.

The qualitative study conducted among health professionals working in these districts suggested that health workers working on plague in these districts had good knowledge of the disease and the measures to be undertaken in case of plague epidemics in these localities. Community health workers, who are volunteers in constant contact with local populations, recognize plague symptoms in villages. The results that we have had are similar to those obtained in other studies conducted in Uganda, where a strong understanding of plague in two plague-endemic areas was explained by health training in drug shops and different sources providing plague information [17].

## Conclusion

The purpose of the current study was to determine the relationship between KAP related to plague of populations, the sociospatial determinants and the epidemiological features in two plague endemic districts of the central highlands in Madagascar. This study has identified that KAP scores related to plague of populations may be influenced by telephone ownership, contact with a former plague, and residence in a municipality with a reported plague case.

The results of this study indicate that, to improve the results in the fight against plague, particularly in prevention, it is necessary to increase the actual KAP levels of populations in plague endemic areas of Madagascar. The contribution of this study was to confirm that a reinforcement of information and communication campaigns is necessary. This goal must be achieved through the allocation of human and financial resources in the priority areas of the central highlands. This allocation of resources should vary at different administrative levels according to their epidemiological contexts.

KAP studies combined with other studies on social and environmental factors would make it possible to assess the roles of social, behavioural and environmental factors in the appearance of human cases of plague and the persistence and re-emergence of epidemics in Madagascar and in regions of the world where plague is still present.

### Recommendations

- Full and detailed information on the plague should be included in the school curriculum in Madagascar. Especially in endemic areas.
- The use of technology, especially mobile phones, should be promoted during awareness campaigns.
- Capacity building of health professionals and community workers on the plague should be a priority, especially in endemic areas.

## Supplementary Information

**Additional file 1.** KAP Related to Plague Questionnaire, Individual KAP Questionnaire administered.

## Data Availability

The datasets used and/or analyzed during the current study are available from the corresponding author on reasonable request.

## Abbreviations

CSB: Centre de Santé de Base (Basic Health Centre)
OR: Odds Ratio
KAP: Knowledge, Attitudes and Practices
SR: Sex Ratio
