# Supplementary material for: Influence of Sociospatial determinants on knowledge, attitudes and practices related to the plague in a population living in endemic areas in the central highlands, Madagascar
Source: BMC Public Health. 2021 Jun 9;21:1102. doi: 10.1186/s12889-021-11101-3 (PMC8191115; doi:10.1186/s12889-021-11101-3)
Supplement: Supplementary file 1 — Additional file 1. KAP Related to Plague Questionnaire, Individual KAP Questionnaire administered. [file 12889_2021_11101_MOESM1_ESM.docx]

| **Knowledge, Attitudes and Practices Related to the Plague in a Population Living in** |
| --- |
| **Endemic Areas in the Central Highlands** |
| **QUESTIONNAIRE** |
| **GPS Coordinates \|_\|_\|. \|_\|_\|\|_\|** |
| **Municipality \|_\|_\|\|_\|** |
| **District \|_\|_\|\|_\|** |
| **Region \|_\|_\|\|_\|** |
| **Household code \|_\|_\|** |
| **Individual Code \|_\|_\|** |
| **Date of visit \|_\|_\|\|_\|_\|\|_\|_\|** |

| **Q.** | | | | **I- INDIVIDUAL INFORMATIONS** | | | |  | | | |
| --- | --- | --- | --- | --- | --- | --- | --- | --- | --- | --- | --- |
| **I.1** | | | | **Name:** | | | |  | | | |
| **I.2** | | | | **First name:** | | | |  | | | |
| **I.3** | | | | **Age:** | | | |  | | | |
| **I.4** | | | | **Sex** | | | |  | | | |
|  | | | | 1= Male | | | |  | | | |
|  | | | | 2= Female | | | |  | | | |
| **I.5** | | | | **Marital status** | | | |  | | | |
|  | | | | 1= Single | | | |  | | | |
|  | | | | 2= Married | | | |  | | | |
|  | | | | 3= Divorced | | | |  | | | |
|  | | | | 4= Widowed | | | |  | | | |
| **I.6** | | | | **Profession:** | | | |  | | | |
|  | | | |  | | | |  | | | |
| **I.7 a** | | | | **Are you a current student?** | | | |  | | | |
|  | | | | 1= Yes | | | |  | | | |
|  | | | | 2= No | | | |  | | | |
| **I.7 b** | | | | **What level of education have you achieved?** | | | |  | | | |
|  | | | | 1= No | | | |  | | | |
|  | | | | 2= Primary school | | | |  | | | |
|  | | | | 3= Secondary school | | | |  | | | |
|  | | | | 4= University | | | |  | | | |
|  | | | |  | | | |  | | | |
| **I.8** | | | | **Do you have tools to access the information?** | | | |  | | | |
|  | | | | 1= Yes | | | |  | | | |
|  | | | | 2= No | | | |  | | | |
|  | | | |  | | | |  | | | |
| **I.9** | | | | **What kind of tools do you have to access the information?** | | | |  | | | |
|  | | | | 1= Radio | | | |  | | | |
|  | | | | 2= TV | | | |  | | | |
|  | | | | 3= Newspaper | | | |  | | | |
|  | | | | 4= Other | | | |  | | | |
|  | | | |  | | | |  | | | |
| **I.10** | | | | **Which means do you use for moving and/or travelling?** | | | |  | | | |
|  | | | | 1= By foot | | | |  | | | |
|  | | | | 2= Plough | | | |  | | | |
|  | | | | 3= Car | | | |  | | | |
|  | | | | 4= Bicycle | | | |  | | | |
|  | | | | 5= Motorcycle | | | |  | | | |
|  | | | | 6= Public transport | | | |  | | | |
|  | | | | 7= Other | | | |  | | | |
|  | | | |  | | | |  | | | |
| **I.11** | | | | **Have you ever had contact with a former plague case?** | | | |  | | | |
|  | | | | 1= Yes | | | |  | | | |
|  | | | | 2= No | | | |  | | | |
|  | | | | 3= Don't know | | | |  | | | |
|  | | | |  | | | |  | | | |
| **I.12** | | | | **If so, specify?** | | | |  | | | |
|  | | | | 1= A family member | | | |  | | | |
|  | | | | 2= An acquaintance | | | |  | | | |
|  | | | | 3= A neighbour | | | |  | | | |
|  | | | | 4= Other | | | |  | | | |
|  | | | **II- KNOWLEDGE ABOUT PLAGUE** | | | |  | | | |  |
| **II.1** | | | **Have you ever heard about plague?** | | | |  | | | |  |
|  | | | 1= Yes | | | |  | | | |  |
|  | | | 2= No | | | |  | | | |  |
|  | | | 3= Don't know | | | |  | | | |  |
|  | | |  | | | |  | | | |  |
| **II.2** | | | **How did you hear about it?** | | | |  | | | |  |
|  | | | 1= At school | | | |  | | | |  |
|  | | | 2= Awareness campaign | | | |  | | | |  |
|  | | | 3= On the radio | | | |  | | | |  |
|  | | | 4= Neighbour | | | |  | | | |  |
|  | | | 5= Posters | | | |  | | | |  |
|  | | | 6= Other | | | |  | | | |  |
|  | | |  | | | |  | | | |  |
| **II.3** | | | **Do you think you have enough information about the plague?** | | | |  | | | |  |
|  | | | 1= Yes | | | |  | | | |  |
|  | | | 2= No | | | |  | | | |  |
|  | | | 3= Don't know | | | |  | | | |  |
|  | | |  | | | |  | | | |  |
| **II.4** | | | **How many types or clinical forms of plague do you know?** | | | |  | | | |  |
|  | | |  | | | |  | | | |  |
|  | | |  | | | |  | | | |  |
| **II.5** | | | **Which ones?** | | | |  | | | |  |
|  | | | 1= Bubonic plague | | | |  | | | |  |
|  | | | 2= Pneumonic plague | | | |  | | | |  |
|  | | | 3= Septicaemic plague | | | |  | | | |  |
|  | | | 4= Don't know | | | |  | | | |  |
|  | | |  | | | |  | | | |  |
| **II.6** | | | **What symptoms of the plague do you know?** | | | |  | | | |  |
|  | | | 1= Fever | | | |  | | | |  |
|  | | | 2= Buboes | | | |  | | | |  |
|  | | | 3= Cough | | | |  | | | |  |
|  | | | 4= Headache | | | |  | | | |  |
|  | | | 5= Thrills | | | |  | | | |  |
|  | | | 6= Nausea | | | |  | | | |  |
|  | | | 7= Barfing | | | |  | | | |  |
|  | | |  | | | |  | | | |  |
| **II.7** | | | **Is plague contagious?** | | | |  | | | |  |
|  | | | 1= Yes | | | |  | | | |  |
|  | | | 2= No | | | |  | | | |  |
|  | | | 3= Don't know | | | |  | | | |  |
|  | | |  | | | |  | | | |  |
| **II.8** | | | **How do you think the plague is transmitted?** | | | |  | | | |  |
|  | | | 1= Flea bite | | | |  | | | |  |
|  | | | 2= Airborne transmission | | | |  | | | |  |
|  | | | 4= Don't know | | | |  | | | |  |
|  | | | 5= Other | | | |  | | | |  |
|  | | |  | | | |  | | | |  |
| **II.9** | | | **Could plague be fatal to humans?** | | | |  | | | |  |
|  | | | 1= Yes | | | |  | | | |  |
|  | | | 2= No | | | |  | | | |  |
|  | | | 3= Don't know | | | |  | | | |  |
|  | | |  | | | |  | | | |  |
| **II.10** | | | **How long do you think it takes for a person to die of the plague** | | | |  | | | |  |
|  | | | **after the onset of signs?** | | | |  | | | |  |
|  | | | Days **\|_\|_\|** | | | |  | | | |  |
|  | | | Weeks **\|_\|_\|** | | | |  | | | |  |
|  | | | Months **\|_\|_\|** | | | |  | | | |  |
|  | | | Years **\|_\|_\|** | | | |  | | | |  |
|  | | |  | | | |  | | | |  |
| **II.11** | | | **How long do you think it takes for a person to die of the plague,** | | | |  | | | |  |
|  | | | **without treatment, after the onset of signs?** | | | |  | | | |  |
|  | | | Days **\|_\|_\|** | | | |  | | | |  |
|  | | | Weeks **\|_\|_\|** | | | |  | | | |  |
|  | | | Months **\|_\|_\|** | | | |  | | | |  |
|  | | | Years **\|_\|_\|** | | | |  | | | |  |
|  | | |  | | | |  | | | |  |
| **II.12** | | | **Are there any remedies or medicines for plague?** | | | |  | | | |  |
|  | | | 1= Yes | | | |  | | | |  |
|  | | | 2= No | | | |  | | | |  |
|  | | | 3= Don't know | | | |  | | | |  |
|  | | |  | | | |  | | | |  |
| **II.13** | | | **If yes, which ones?** | | | |  | | | |  |
|  | | | 1= Tablets | | | |  | | | |  |
|  | | | 2= Antibiotics | | | |  | | | |  |
|  | | | 3= Injection | | | |  | | | |  |
|  | | | 4= Herbal tea | | | |  | | | |  |
|  | | | 5= Other | | | |  | | | |  |
|  | | | **If other, specify :…………………………………………………………….** | | | |  | | | |  |
|  | | |  | | | |  | | | |  |
| **II.14** | | | **Where can you find these remedies/medicines against the plague?** | | | |  | | | |  |
|  | | | 1= Health facility | | | |  | | | |  |
|  | | | 2= Hospital | | | |  | | | |  |
|  | | | 3= Traditional practitioner (Traditional herbal tea provider) | | | |  | | | |  |
|  | | | 4= Drugstore | | | |  | | | |  |
|  | | | 5= Traditional healer | | | |  | | | |  |
|  | | | 6= Other | | | |  | | | |  |
|  | | **III- ATTITUDES TOWARDS OTHERSIN CASE OF ILLNESS AND PERCEPTION OF PLAGUE** | | | |  | | | |  |  |
| **III.1** | | **If you are ill, or worry about onset of a disease symptom,** | | | |  | | | |  |  |
|  | | **do you usually share it with your circle?** | | | |  | | | |  |  |
|  | | 1= Yes | | | |  | | | |  |  |
|  | | 2= No | | | |  | | | |  |  |
|  | | 3= Don't know | | | |  | | | |  |  |
|  | |  | | | |  | | | |  |  |
| **III.2** | | **When do you share it with your circle?** | | | |  | | | |  |  |
|  | | 1= In case of severe fever | | | |  | | | |  |  |
|  | | 2= In case of diarrhea. | | | |  | | | |  |  |
|  | | 3= In case of loss of consciousness | | | |  | | | |  |  |
|  | | 4= In case of malaise | | | |  | | | |  |  |
|  | | 5= If you have a headache | | | |  | | | |  |  |
|  | | 6= If you develop pimples on your body | | | |  | | | |  |  |
|  | | 7= In case of severe pain | | | |  | | | |  |  |
|  | | 8= If there are convulsions | | | |  | | | |  |  |
|  | | 9= Don't know | | | |  | | | |  |  |
|  | | 10= Other | | | |  | | | |  |  |
|  | |  | | | |  | | | |  |  |
| **III.3** | | **In case of illness, would you take specific measures to protect those around you?** | | | |  | | | |  |  |
|  | | 1= Yes | | | |  | | | |  |  |
|  | | 2= No | | | |  | | | |  |  |
|  | | 3= Don't know | | | |  | | | |  |  |
|  | |  | | | |  | | | |  |  |
| **III.4** | | **If so, which of the following would you adopt?** | | | |  | | | |  |  |
|  | | 1= Give medication to the family and friends | | | |  | | | |  |  |
|  | | 2= Consult the nearest doctor | | | |  | | | |  |  |
|  | | 3= Go to a health facility | | | |  | | | |  |  |
|  | | 4= Isolate yourself | | | |  | | | |  |  |
|  | | 5= Do nothing | | | |  | | | |  |  |
|  | | 6= Use of herbal teas or other medicinal plants | | | |  | | | |  |  |
|  | | 7= Don't know | | | |  | | | |  |  |
|  | | 8= Other | | | |  | | | |  |  |
|  | |  | | | |  | | | |  |  |
| **III.5** | | **According to you, which of the following statements are correct?** | | | |  | | | |  |  |
|  | | 1= It is a serious disease | | | |  | | | |  |  |
|  | | 2= It is a shameful disease | | | |  | | | |  |  |
|  | | 3= It is a divine disease | | | |  | | | |  |  |
|  | | 4= It is a deadly disease | | | |  | | | |  |  |
|  | | 5= It is the consequence of uncleanliness | | | |  | | | |  |  |
|  | | 6= It is the disease of the rat | | | |  | | | |  |  |
|  | | 7= It is due to a transgressed taboo | | | |  | | | |  |  |
|  | | 8= Don't know | | | |  | | | |  |  |
|  | | 10= Other | | | |  | | | |  |  |
|  | **IV- PRACTICES FOR SEEKING CARE IN CASES OF PLAGUE OR OTHER DISEASES** | | | |  | | | |  |  |  |
| **IV.1** | **What type of health structure do you consult in case of illness?** | | | |  | | | |  |  |  |
|  | 1= Health facility | | | |  | | | |  |  |  |
|  | 2= Hospital | | | |  | | | |  |  |  |
|  | 3= Traditional healer | | | |  | | | |  |  |  |
|  | 4= Other | | | |  | | | |  |  |  |
|  |  | | | |  | | | |  |  |  |
| **IV.2** | **If disease is suspected:** | | | |  | | | |  |  |  |
| **a** | **When do you go to the CSB?** | | | |  | | | |  |  |  |
|  |  | | | |  | | | |  |  |  |
| **b** | **When do you go to the hospital?** | | | |  | | | |  |  |  |
|  |  | | | |  | | | |  |  |  |
| **c** | **When do you consult a traditional healer?** | | | |  | | | |  |  |  |
|  |  | | | |  | | | |  |  |  |
| **IV.3** | **In case of signs that might suggest plague (fever, buboes...), would you go to see a doctor?** | | | |  | | | |  |  |  |
|  | 1= Yes | | | |  | | | |  |  |  |
|  | 2= No | | | |  | | | |  |  |  |
|  | 3= Don't know | | | |  | | | |  |  |  |
|  |  | | | |  | | | |  |  |  |
| **IV.4** | **Have you ever sought health facility after experiencing** | | | |  | | | |  |  |  |
|  | **plague-like symptoms?** | | | |  | | | |  |  |  |
|  | 1= Yes | | | |  | | | |  |  |  |
|  | 2= No | | | |  | | | |  |  |  |
|  | 3= Don't know | | | |  | | | |  |  |  |
